# Supplementary figures and images for: l-Malate (−2) Protonation State is Required for Efficient Decarboxylation to l-Lactate by the Malolactic Enzyme of Oenococcus oeni
Source: Molecules. 2020 Jul 28;25(15):3431. doi: 10.3390/molecules25153431 (PMC7435853; doi:10.3390/molecules25153431)

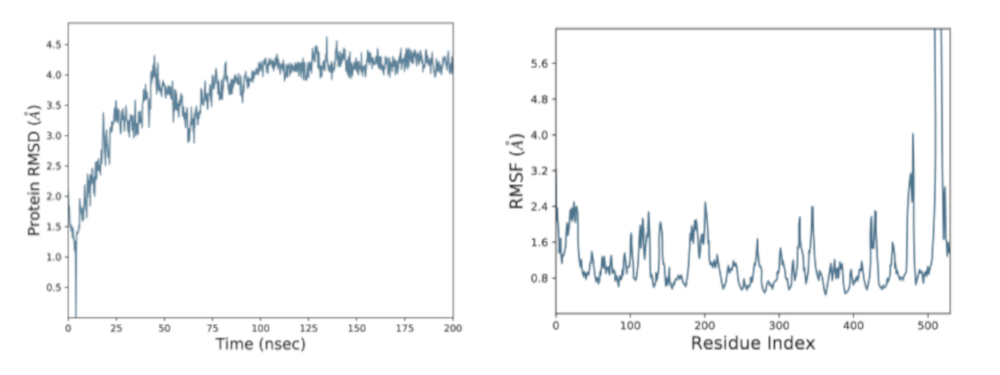

Supplement: Supplementary file 1 [file molecules-25-03431-s001.zip › molecules-837718-supplementary-proof-change/Supplementary files Acevedo et al.2020/Figure S1.tif]

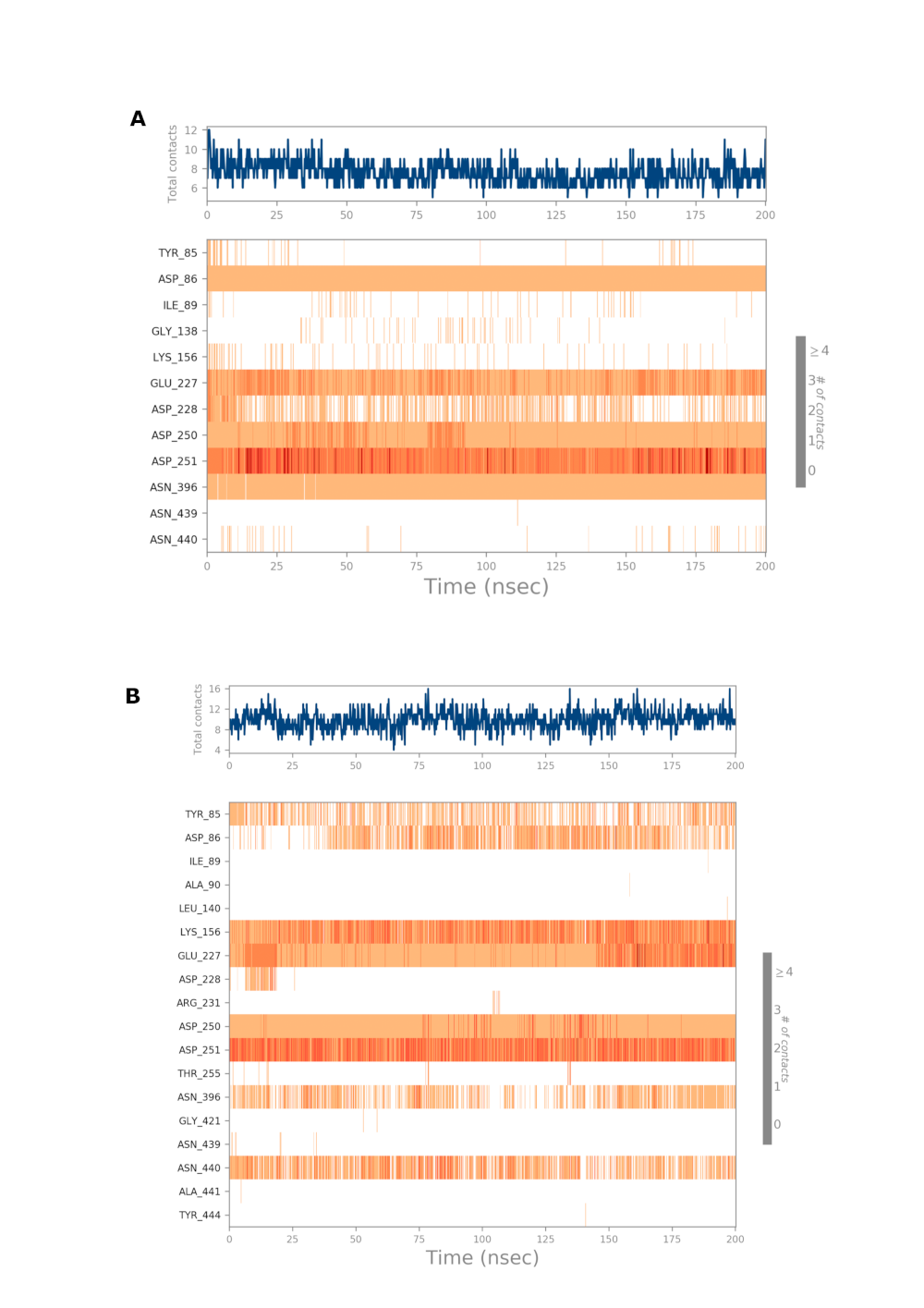

Supplement: Supplementary file 1 [file molecules-25-03431-s001.zip › molecules-837718-supplementary-proof-change/Supplementary files Acevedo et al.2020/Figure S2.tif]

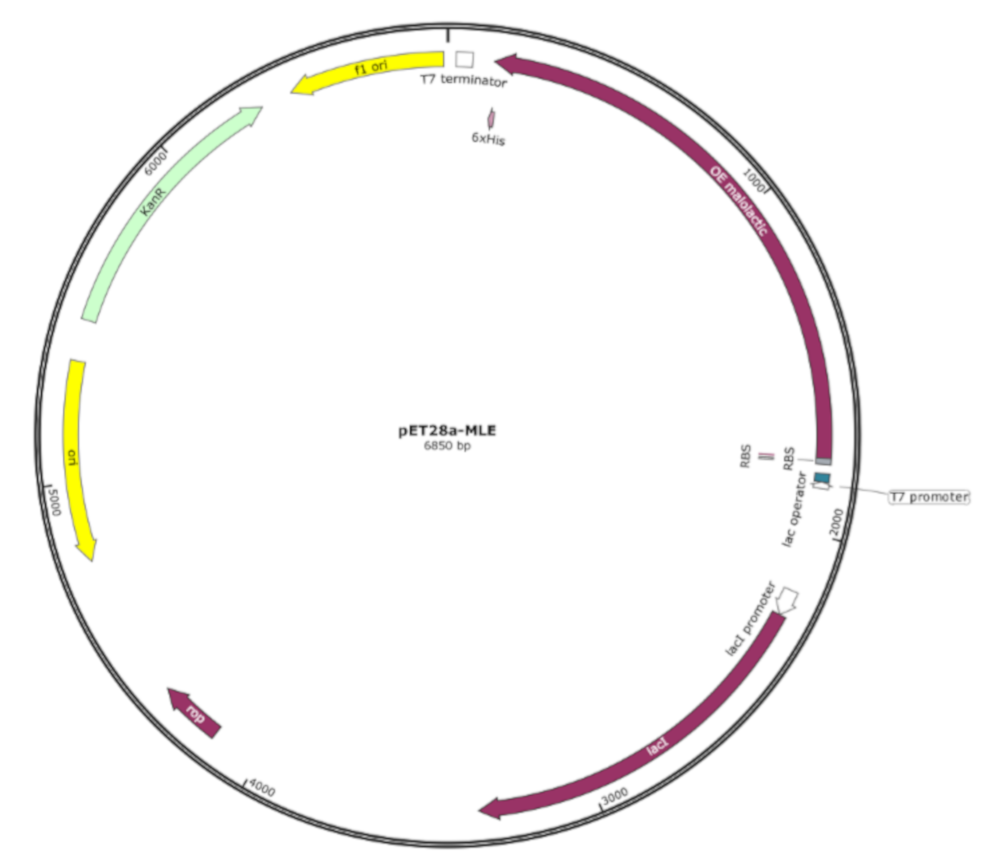

Supplement: Supplementary file 1 [file molecules-25-03431-s001.zip › molecules-837718-supplementary-proof-change/Supplementary files Acevedo et al.2020/Figure S3.tif]
